# Altered NK cell function in obese healthy humans

**DOI:** 10.1186/s40608-014-0033-1

**Published:** 2015-01-24

**Authors:** Tobias Laue, Christiane D Wrann, Birgit Hoffmann-Castendiek, Daniel Pietsch, Lena Hübner, Heike Kielstein

**Affiliations:** Institute for Functional and Applied Anatomy, Hannover Medical School, Hannover, Germany; Centre for Pediatrics and Adolescent Medicine, Hannover Medical School, Hannover, Germany; Dana-Farber Cancer Institute, Beth Israel Deaconess Medical Center, Harvard Medical School, Boston, MA USA; Clinic for Pneumology, Hannover Medical School, Hannover, Germany; Institute of Clinical Chemistry, Hannover Medical School, Hannover, Germany; Department of General-, Visceral-, Vascular and Thoracic Surgery, Charité, University Medicine Berlin, Campus Mitte, Berlin, Germany; Department of Anatomy and Cell Biology, Martin Luther University Halle-Wittenberg, Halle (Saale), Germany

**Keywords:** Obesity, Immunity, Natural killer (NK) cells, Leptin, Ob-R, TRAIL, CD107a

## Abstract

**Background:**

Obesity is associated with an elevated risk for several types of cancer and thus a major health hazard. However, the mechanism between overweight and cancer susceptibility is still elusive. Leptin, mainly produced by adipocytes links food intake and energy expenditure. In addition, recent studies have shown an immunomodulatory impact of leptin on NK cells. The purpose of the present study was to investigate whether leptin stimulation of NK cells from obese humans leads to altered functions as compared to NK cells from lean subjects. On the basis of body mass index 20 healthy individuals were classified in two groups: normal weight (<25 kg/m^2^) and obese (>30 kg/m^2^). Peripheral blood mononuclear cells (PBMC) were isolated from blood samples. We used flow cytometry to assess differences in phenotype and activity markers (CD107a, CD178 and TRAIL) of PBMCs between both groups. Furthermore, we determined after short-term *in vitro* leptin stimulation the phosphorylation of JAK2, downstream target of the intracellular signaling cascade of the leptin receptor, by Western Blotting and numbers of NK-cell-tumor-cell-conjugates as well as Granzyme^+^ and IFN-γ^+^ NK cells by flow cytometry. Finally, the proliferative capacity of control and long-term (7 days) leptin-stimulated NK cells was examined.

**Results:**

As opposed to similar NK cell counts, the number of CD3^+^CD56^+^ cells was significantly lower in obese compared to lean subjects. Human NK cells express the leptin receptor (Ob-R). For further determination of Ob-R, intracellular target proteins of PBMCs were investigated by Western Blotting. Phosphorylation of JAK2 was lower in obese as compared to normal weight subjects. Furthermore, significantly lower levels of TNF-related apoptosis-inducing ligand (TRAIL) as an NK cell functional marker in obese subjects were found. *In vitro* leptin stimulation resulted in a higher production of interferon-γ in NK cells of normal weight subjects. Interestingly, long-term leptin stimulation had no significant influence on numbers of proliferating NK cells.

**Conclusions:**

NK cells from obese healthy humans show functional deficits and altered responses after *in vitro* leptin challenge.

## Background

Obesity is a major health hazard manifested by its rapidly increasing prevalence [1]. Beside a reduced life expectancy, obesity is associated with an elevated risk for certain diseases like cardiovascular disease and type 2 diabetes [2] as well as severe inflammations [3]. Furthermore, obese individuals have a higher risk for several types of cancer, e.g. oesophageal adenocarcinoma, postmenopausal breast cancer and colon cancer [4,5]. Several mechanisms behind the link between an increased bodyweight and cancer risk are actually subject of research: the insulin-IGF (insulin-like growth factor) axis [6,7], increased bioavailability of steroid hormones [8] and adipose tissue-derived hormones and cytokines (adipokines) [9,10].

One of these adipocyte-derived hormones is the 16-kDa leptin [11], mainly produced by the white adipose tissue. The serum levels of leptin the product of the obese (ob) gene, correlate with body weight and is higher in obese individuals [12]. There are at least six alternatively spliced isoforms of the leptin receptor (Ob-Ra – Ob-Rf). Beside Ob-Re, the soluble form, all leptin-receptors share an intracellular binding site for the receptor-associated Janus-family tyrosine kinase-(JAK) 2. However, only the long-form, Ob-Rb, is capable in mediating all leptin actions [13]. Upon ligand binding to Ob-Rb, JAK2 becomes auto-phosphorylated and promotes the phosphorylation of three tyrosine residues (Tyr985, Tyr1077 and Tyr1138) on the intracellular domain of Ob-Rb for distinct signaling pathways [14]. Via JAK2 phosphorylated Tyr1183 recruits the signal transducer and activator of transcription (STAT) 3 for the JAK/STAT-pathway. Activated STAT3 translocates to the nucleus for the regulation of gene expression.

Ob-R, product of the db (diabetes) gene, is localized in several tissues mediating transport and degradation of leptin but only the long-form is highly expressed in the hypothalamus [15]. Moreover, leptin-receptor-deficient (db/db) mice share an abnormally spliced leptin-receptor resulting in a phenotype of severely obese animals [16]. Accordingly, leptin links the nutritional status by acting in hypothalamic nuclei and regulating food intake and energy expenditure [17].

However, in the last years it becomes evident that leptin also affects both the adaptive and the innate immune system [18,19]. Natural killer (NK) cells belong with their abilities in production of cytokines, such as interferon-γ (IFN-γ), and cytotoxicity against transformed as well as infected cells to the innate immunity. Beside a variety of activating and inhibitory receptors, NK cells express Ob-R and have increased cytotoxicity after leptin stimulation in vitro [20]. However, a recent study of our group showed diminished immune functions after long-term leptin exposure of human NK cells [21]. Db/db mice have an impaired NK cell activity suggesting leptin as necessary for the development and activation of NK cells [22]. An 11-year-follow-up study indicated that low cytotoxic activity of peripheral-blood lymphocytes is associated with increased cancer susceptibility [23]. Our group demonstrated attenuated NK cell activity in diet-induced obese rats after leptin administration caused through abrogated post-receptor signaling of the JAK/STAT-pathway [24]. However, only few data exist describing the activity of NK cells in obese compared to normal weight humans. In the present study, we observed lower activity of NK cells as well as significantly lower levels of components of the Ob-R signaling pathway in obese healthy humans. Furthermore, NK cells in obese showed a non-significant altered proliferation process suggesting leptin as a possible link between bodyweight, lower NK cell functionality and herewith increased cancer susceptibility in obese humans.

## Methods

### Study subjects

The study was approved by the ethics committee of the Hannover Medical School, Hannover, Germany. Informed consent has been received from 20 healthy subjects. All subjects’ data were determined by self-report and measured with a standardized questionnaire. Exclusion criteria were an age < 18 or > 65 years, an acute infection, immunosuppression or known cancer in anamnesis. On the basis of body mass index (BMI; kg/m^2^) the study subjects were classified in two groups: obese with BMI > 30 kg/m^2^ (3 females and 9 males) and normal weight with BMI < 25 kg/m^2^ (4 females and 4 males). All obese subjects were patients in the sleep laboratory of Hannover Medical School for medical examination of sleep apnea syndrome.

### Isolation of human peripheral blood mononuclear cells (PBMC) from human subjects and measurement of triglyceride, cholesterol and glucose

Blood samples were taken from the study subjects between 8 a.m. and 10 a.m. and immediately heparinized. A small sample from 10 subjects (normal weight: 5; obese: 5) was used to measure levels of triglyceride, cholesterol and glucose via photometry by P800 module of Roche MODULAR PPE (Roche and Hitachi, Japan). Furthermore, the blood was diluted with phosphate buffered saline (PBS) and the peripheral blood mononuclear cells (PBMC) were isolated from cell suspension by Ficoll gradient (Biocoll, Biochrom, Berlin, Germany) and collected from the interphase.

### Reagents

Recombinant human leptin was obtained from R&D Systems (Wiesbaden, Germany) and diluted to 50 nM.

### Flow cytometry

For phenotypic analyses PBMCs were stained with the directly labelled monoclonal mouse-anti human antibodies CD3 conjugated with phycoerythrin (PE)-Cy7 (CD3-PE-Cy7) (clone SK7, 1:50), CD56 conjugated with allophycocyanin (CD56-APC) (clone NCAM16.2, 1:100), anti-hLeptin R (Ob-R) conjugated with carboxyfluorescein (anti-hLeptin R-CFS) (clone 52263, 1:20) (R&D Systems, Wiesbaden, Germany), CD253-PE (TRAIL; clone RIK-2, 1:20) (BD Biosciences, San Diego, CA), CD107a conjugated with FITC (clone H4A3, 1:10) (BD Biosciences), and biotinylated CD178 (clone NOK-1, 1:10) (BD Biosciences) followed by labeling with PerCP-Cy5.5-conjugated streptavidin (1:500) (BD Pharmingen, Heidelberg, Germany). PBMCs (5 × 10^5^ cells/100 μl) were incubated in 96 well-round bottom plates with the above mentioned antibodies for 20 min at 4°C, washed twice with measuring buffer and analyzed by flow cytometry using a FACSCanto (BD Biosciences, San Jose, CA) with FACS Diva software v5.0.3. A well with cells stained with the above mentioned antibodies except for anti-hLeptin R-CFS served as control for the measurements of Leptin R.

### Intracellular staining

After 24 h of cell culture and stimulation with leptin (50nM; R&D Systems) the expression of intracellular cytokines by NK cells was analyzed using a FACSCanto cytometer. Prior to intracellular labelling cell surface staining was performed. PBMCs (5 × 10^5^ cells/100 μl in 96-well round-bottom plates) were stained with CD3-PE (1:250) and CD56-APC (1:100). After 15 min at 4°C, cells were washed twice with measuring buffer and incubated with PBS supplemented with 4% paraformaldehyde (Merck, Darmstadt, Germany) in the dark for 10 min at room temperature. Cells were washed twice with measuring buffer and centrifuged for 3 min at 400 g. Thereafter cells were washed once with saponin buffer (aqua dest. supplemented with 0.1% saponin and 0.01 M HEPES), then resuspended in saponin buffer and stained with the directly labelled mononuclear mouse anti-human antibodies granzyme A (GzmA) conjugated with fluorescein isothiocyanate (GzmA-FITC) (clone CB9, 1:100) and IFN-γ-PE-Cy7 (clone 4S.B3, 1:100) (both BD Biosciences). To prevent non-specific binding via Fc receptors, each well was supplemented with 5 μl Pentaglobin. Finally cells were washed three times and centrifuged for 3 min at 400 g. The data from flow cytometric analyses were processed with FACS Diva software v5.0.3. A well with cells stained with the above mentioned antibodies except for IFN-γ-PE-Cy7 served as control for the measurements of IFN-γ expression by NK cells.

### Conjugate-forming assay

After two days of culture and 24 h *in vitro* stimulation with leptin (50 nM) or vehicle in R10 medium (containing 10% FCS, 100 U/ml penicillin, 100 μg/ml streptomycin) cell surface staining of the PBMCs (5 × 10^5^ cells/100 μl) was performed in 96-well round-bottom plates by adding CD3-PE (1:250) and CD56-APC (1:100) and incubating for 15 min at 4°C. After two washes and a centrifugation (400 g for 3 min), each well was supplemented with 1×10^6^/ml cells of the K562 erythroleukemia line (which were maintained in suspension culture flasks at 37°C in a humidified atmosphere with 5% CO_2_). Cells were centrifuged at 100 g for 3 min at 4°C and incubated for 15 min at 37°C, 5% CO_2_ and 85% RH. Cells were carefully resuspended and transferred into FACS tubes using pipet tips with expanded apertures. After gently mixing the cells, conjugate formation was analyzed using a FACSCanto (BD Biosciences) by gating on PBMC and K562 cells, excluding CD3^+^ T cells.

### Proliferation assay

PBMCs (0.5 × 10^6^ cells / 250 μl) were incubated with CFSE (Carboxy Fluorescein Succinimidyl Ester; final concentration 1.5 μM) for 7 min at 37°C in a cell incubator. Cells were resuspended in 250 μl of R10 culture medium (1 × 10^6^/ml) including IL-2 (0.001%) and leptin (50 nM) or vehicle and incubated for 7 days. Medium was refreshed on day 4. After 7 days of culture cell surface staining of PBMCs (5 × 10^5^ cells/100 μl) was performed in 96-well round-bottom plates by adding CD3-PE (1:250) and CD56-APC (1:100) and incubating for 15 min at 4°C. Cells were washed twice with measuring buffer and analyzed by flow cytometry using a FACSCanto (BD Biosciences) with FACS Diva software v5.0.3. The percentage of proliferating cells was determined as the number of gated NK cells that displayed a distinctively lesser fluorescence.

### Western blotting

PBMCs were stained in wells with 1,000,000 cells per well. Negative controls were incubated with PBS whereas the samples were stimulated with 50 nM leptin for 5 and 15 min at 37°C. Stimulated PBMCs were collected on ice, centrifuged (500 g, 3 min, 4°C) and supernatants were removed. Afterwards, the pellets were resuspended in PBS, re-centrifuged and supernatants removed. The pellets were lysed in RIPA buffer (50 mM Tris, pH 7.5, 150 mM NaCl, 0.5% sodium deoxycholate, 1% Nonidet P-40, and 0.1% SDS) containing protease inhibitor (Complete Mini; Roche, Mannheim, Germany), 1 mM sodium orthovanadate, 50 mM NaF, and 200 μg/l okadaic acid for 10 min on ice and stored at −80°C.

Protein concentrations of samples were determined via photometry by Olympus AU400 (Olympus). Equal amounts of protein samples were denatured in 15 μl Laemmli buffer (containing 5% beta mercaptoethanol of total volume; Bio-Rad Laboratories, Hercules, CA) and heated at 95°C for 10 min. The samples were loaded (7 μg per lane) into a NuPAGE 4-12% Bis-Tris gel (Invitrogen, Carlsbad, CA), resolved in sodium dodecyl sulfate-polyacrylamide gel electrophoresis (SDS-PAGE) and transferred onto nitrocellulose membranes with a pore size of 0.2 μm (SERVA Electrophoresis, Heidelberg, Germany).

The membranes were blocked with 5% nonfat milk in Tris-buffered saline (50 mM TrisHCl, pH 7.4 and 150 mM NaCl) containing 0.1% Tween-20 (TBS-T) at room temperature for 60 min on a shaking table, followed by an overnight incubation with primary antibodies in 5% BSA/TBS-T at 4°C on a shaking table. Primary antibodies were anti-phospho-Jak2 (Tyr1007/1008) (dilution 1:1250; New England Biolabs, Frankfurt, Germany) and anti-beta-actin (dilution 1:1250; Sigma-Aldrich, St. Louise, MO). Membranes were rinsed three times (5 min each) with TBS-T, followed by an incubation with horseradish peroxidase-conjugated secondary antibody (dilution 1:3000; New England Biolabs) for 60 min at room temperature. The peroxidase activity on the membrane was visualized on X-ray film by a standard enhanced chemiluminescence (ECL plus; Amersham, GE Healthcare, Freiburg, Germany) procedure. Quantitative analysis of the Western blots of 3 normal weight and 3 obese individuals was performed using ImageJ 1.48v (http://rsb.info.nih.gov/ij). Values were normalized to actin.

### Statistics

Data are expressed as means + SEM. P-values of less then 0.05 were considered significant. The software used was GraphPad Prism 4 (GraphPad Software Inc.). Results were analyzed using one-way ANOVA with the factor ‘body weight’ or ‘stimulation’. If overall effects showed significant differences / interactions Tukey multiple comparison test for post hoc analysis was implemented.

## Results

### BMI and clinical characteristics

In total, 20 subjects were investigated in the study (normal weight: 4 females, 4 males; obese: 3 females, 9 males). No significant differences between the two groups (normal weight and obese) in age and height were found (Table 1). However, the two groups significantly differed in the weight (mean body weight of 70.0 kg vs. 121.6 kg; p < 0.0001) resulting in a significant BMI difference of 22.2 kg/m^2^ vs. 38.5 kg/m^2^ (p < 0.0001). From 10 subjects (normal weight: 5; obese: 5) triglycerides, cholesterol and glucose levels were determined. Concerning triglycerides, cholesterol and glucose levels no significant difference could be detected between normal weight and obese subjects.

**Table 1:** **Baseline characteristics of study population**

	**Normal weight**	**Obese**	**Significance**	**Reference range**
	**(mean ± SEM)**	**(mean ± SEM)**		
Age (years)	39.3 ± 6.4	47.5 ± 3.9	Ø	n.a.
Height (m)	1.77 ± 0.04	1.78 ± 0.03	Ø	n.a.
Weight (kg)	70.0 ± 4.2	121.6 ± 5.3	<0.0001	n.a.
BMI (kg/m^2^)	22.2 ± 0.6	38.5 ± 0.8	<0.0001	n.a.
Triglycerides^§^	1.0 ± 0.3	1.8 ± 0.4	Ø	[0.5–2.95 mmol/l]
Cholesterol^§^	4.4 ± 0.4	4.3 ± 0.3	Ø	[4.1–5.2 mmol/l]
Glucose^§^	4.3 ± 0.3	5.2 ± 0.3	Ø	[3.8–5.5 mmol/l]

SEM = standard error of the mean.

BMI = body mass index.

n.a. = not applicable.

§ = measured for 5 normal weight and 5 obese individuals.

### NK cell numbers

**Figure 1:**
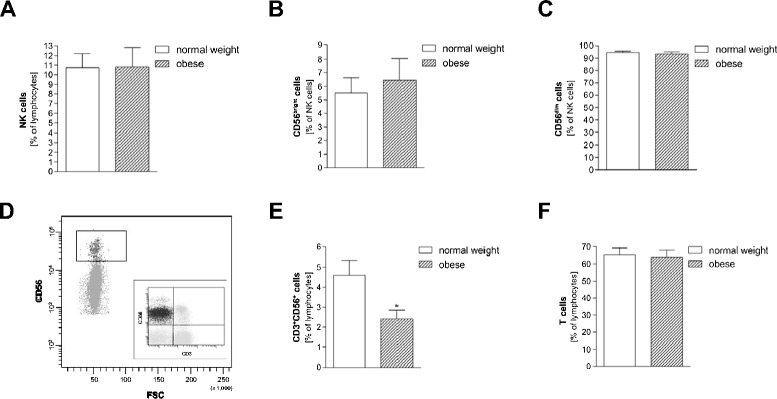
**Leukocyte subsets in healthy normal weight and obese subject** *.* Isolated PBMCs from healthy normal weight and obese subjects were stained with antibodies and analyzed by flow cytometry. **(A)** NK (CD56^+^CD3^−^) cells as percentage of lymphocytes; **(B)** CD56^bright^ cells and **(C)** CD56^dim^ cells as percentage of NK cells. **(D)** Representative flow cytometry dot plots of CD56bright and CD56dim cells from a normal weight individual; insert in the dot plot show the prior gaiting for NK cells (CD56^+^CD3^−^) in the upper left quadrant. **(E)** CD3^+^CD56^+^ cells as percentage of lymphocytes; **(F)** T (CD3^+^CD56^−^) cells as percentage of lymphocytes. Data are expressed as mean + SEM. Significant effects of obese vs. normal weight subjects are indicated by an asterisk *p < 0.05.

In both groups approximately 11% of all lymphocytes were NK cells (Figure 1A). NK cells can be divided in CD56^dim^ and CD56^bright^ by the expression of CD56 [25]. CD56^dim^ NK cells express the surface antigen in low concentration and have the capability to form conjugates with target cells whereas the CD56^bright^ NK cells predominantly produce pro-inflammatory cytokines, e.g. TNF-α and IFN-γ. No significant difference between normal weight and obese concerning the expression of CD56^dim^ and CD56^bright^ was found (Figure 1B-D).

### CD3^+^CD56^+^ cells

CD3^+^CD56^+^ cells have structural similarities with NK cells (CD56; NK1.1) and T cells (CD3; αβ T cell receptors) [26] and are activated by lipid-based antigens presented by CD1d [27]. Obese individuals showed significantly lower amounts of CD3^+^CD56^+^ cells as compared to the normal weight controls (Figure 1E). No distinct difference concerning the T cell numbers from obese and normal weight subjects could be observed (Figure 1F).

### Components of the Ob-R signaling pathway

**Figure 2:**
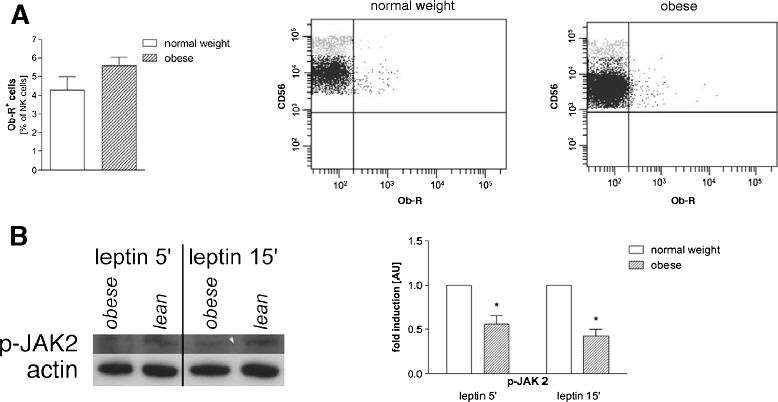
**Leptin receptor (Ob-R) expression and signaling pathway in PBMCs.** PBMCs from healthy normal weight and obese subjects were stained with antibodies and analyzed by flow cytometry; **(A)** Numbers of Ob-R positive NK cells are shown in percentage of NK cells. Data are expressed as mean + SEM. Representative flow cytometry dot plots of ObR+ and ObR- NK cells from a normal weight (middle panel) and an obese individual (right panel) are shown. **(B)** Isolated PBMCs from normal weight and obese individuals (shown with body mass index (BMI)) were stimulated for 5 and 15 min with 50 nM human recombinant leptin. Phosphorylation of JAK2 (p-JAK2) was determined by Western blotting. Actin was used as control for equal loading. The experiment was repeated with 3 normal weight and 3 obese individuals, and a representative Western blot is shown. Quantitative analysis of the Western blots for p-JAK2. Values were normalized to actin and are shown as fold induction in arbitrary units (AU). Data are expressed as mean + SEM. Significant effects of obese vs. normal weight subjects are indicated by an asterisk *p < 0.05.

We determined the Ob-R expression on peripheral blood NK cells with flow cytometry and observed slightly more Ob-R^+^ NK cells in obese subjects (Figure 2A). There are six alternatively spliced Ob-R isoforms sharing the same extracellular domain but only the long form, Ob-Rb, is capable of signal transduction [28]. To evaluate the signal transduction via Ob-Rb we determined the phosphorylation of JAK2 in isolated PBMCs after *in vitro* stimulation with leptin for 5 and 15 min by Western blotting. Lower levels of p-JAK2, a downstream target of the intracellular signaling cascade of the Ob-Rb receptor, were detected PBMCs in obese subjects (Figure 2B).

### TRAIL, CD107a and CD178 expression by NK cells

**Figure 3:**
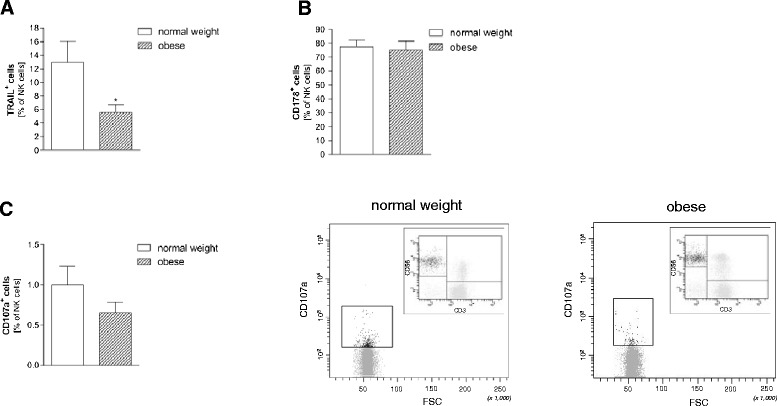
**Expression of functional parameters of NK cells** *.* PBMCs were stained with antibodies and analyzed by flow cytometry to evaluate the differences of functional parameters in normal weight and obese subjects. **(A)** TNF-related apoptosis-inducing ligand (TRAIL) positive NK cells, **(B)** CD178 positive NK cells, and **(C)** CD107a positive NK cells as percentage of NK cells. Representative flow cytometry dot plots of CD107a from a normal weight (middle panel) and an obese individual (right panel). Inserts in both dot plots show the prior gating for NK cells (CD56^+^CD3^−^) in the upper left quadrant. Data are expressed as mean + SEM. Significant effects of obese vs. normal weight subjects are indicated by an asterisk *p < 0.05.

To evaluate differences in the activity of human NK cells in obese and normal weight subjects we measured the expression of TNF-related apoptosis-inducing ligand (TRAIL), CD107a and CD178 by flow cytometry. Surface-bound TRAIL is well-known as one effector mechanism of NK cells to induce apoptosis [29,30]. Compared to normal weight subjects the TRAIL positive NK cells were significantly lower in the obese (Figure 3A). No difference between normal weight and obese subjects could be observed concerning the number of CD178 (Fas ligand; a crucial promoter for programmed cell death by apoptosis; [31,32]) expressing NK cells (Figure 3B).

The number of NK cells expressing CD107a, another marker of NK cell activity (e.g. for IFN-γ and TNF-α cytokine production; [33]), were reduced by nearly 40% in the obese subjects as compared to the lean controls (Figure 3C).

### Intracellular IFN-γ expression

**Figure 4:**
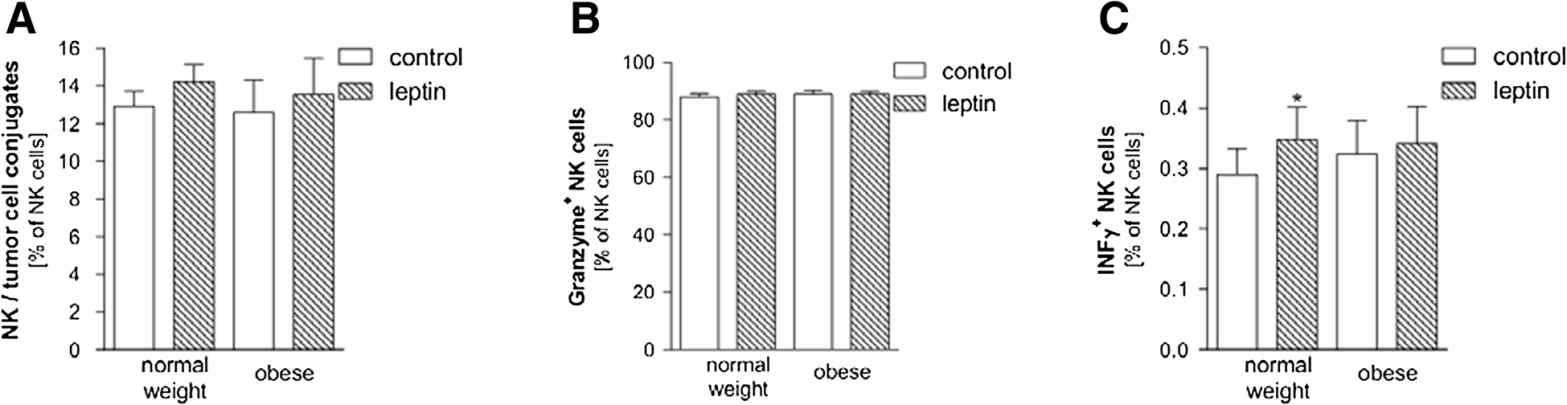
**Effects of a leptin stimulation on NK-cell-tumor-cell-conjugates and IFN-γ and Granzyme production of human NK cells.** PBMCs from healthy normal weight and obese subjects were stimulated with 50 nM human recombinant leptin or treated with vehicle for 24 h. Thereafter, cells were stained with antibodies and analyzed by flow cytometry. **(A)** Numbers of NK-cell-tumor-cell-conjugates (K562 erythroleukemia line), **(B)** Granzyme positive NK, **(C)** IFN-γ positive NK cells are shown as percentage of NK cells. Data are expressed as mean + SEM. Significant effects of leptin stimulated IFN-γ positive vs. non-stimulated NK cells in the normal weight group are indicated by an asterisk *p < 0.05.

To evaluate the influence of a leptin stimulation on different functional parameters, human NK cells were stimulated with human recombinant leptin for 24 h. Numbers of NK-cell-tumor-cell-conjugates as well as Granzyme positive and IFN-γ positive NK cells were determined by flow cytometry. Short-term leptin administration resulted in slightly improved levels of NK-cell-tumor-cell-conjugates as compared to the vehicle treated controls both in normal weight and obese subjects (Figure 4A). NK cells induce cell death via Granzymes released into target cells [34,35]. Leptin stimulation was without effect on the numbers of Granzyme^+^ NK cells (Figure 4B). However, the intracellular IFN-γ expression after leptin administration was significantly higher in normal weight subjects as compared to the corresponding normal weight controls (Figure 4C). No stimulation effect could be seen in the NK cells from obese subjects.

### NK cell proliferation after long-term leptin stimulation

**Figure 5:**
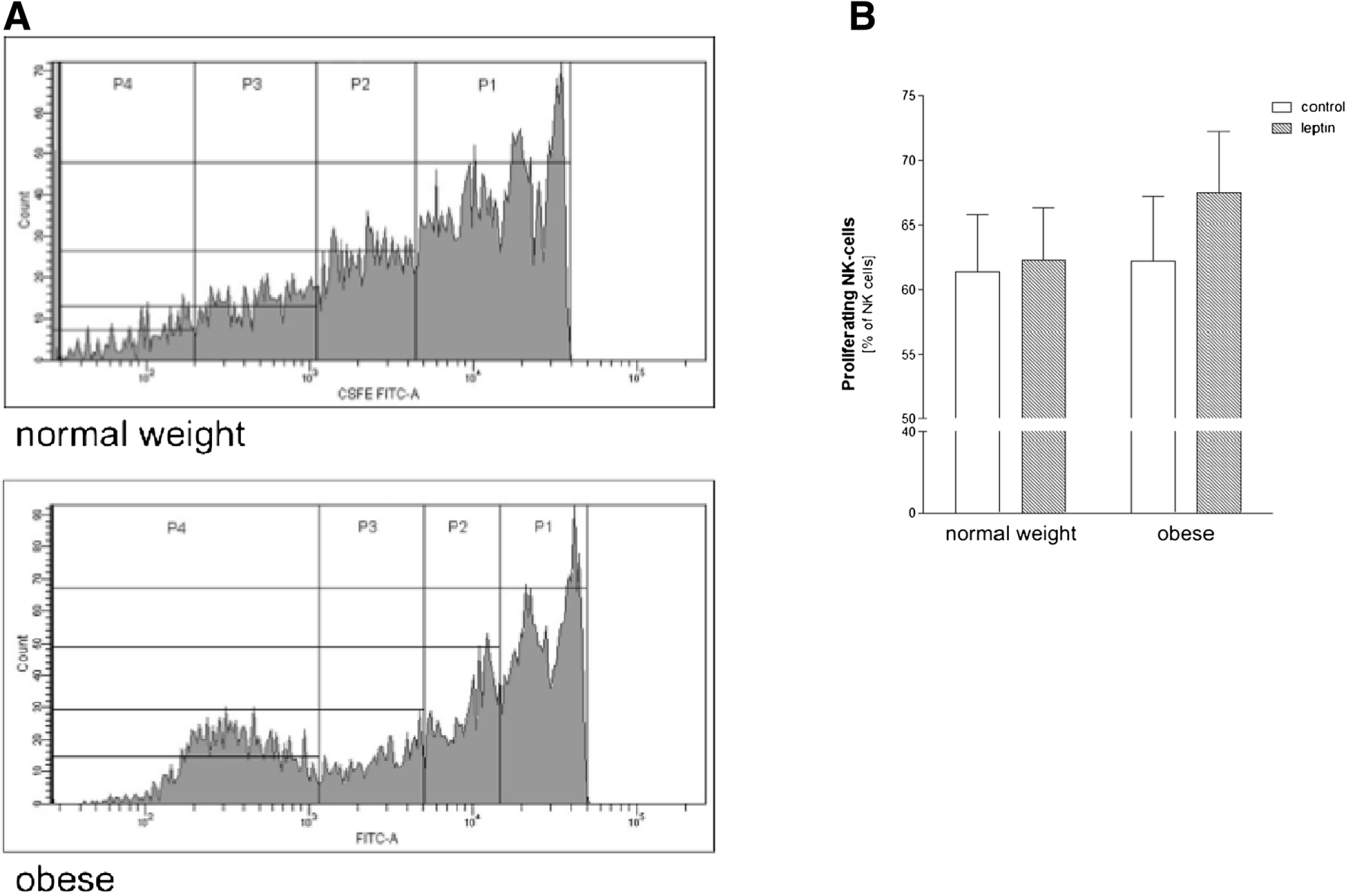
**Effect of leptin stimulation on cell proliferation of human NK cells** *.* PBMCs from healthy normal weight and obese subjects were stimulated with 50 nM human recombinant leptin or vehicle for 7 days, stained with antibodies and analyzed with flow cytometry. **(A)** Representative histograms of CSFE staining showing four proliferation peaks of NK cells from a normal weight (upper histogram) and an obese (lower histogram) subjects. The peaks, from P4 (parental generation) to P1, represent successive generations. **(B)** Numbers of proliferating NK cells as percentage of NK cells. Data are expressed as mean + SEM.

To evaluate the proliferative capacity of control and long-term (7 days *in vitro*) leptin stimulated NK cells in normal weight and obese subjects levels of proliferating NK cells were determined by flow cytometry. No significant difference could be detected between numbers of proliferating NK cells in obese subjects as compared to corresponding normal weight (~60% of all NK cells; Figure 5B). Long-term leptin stimulation resulted in a non-significant higher amount of proliferating NK cells in obese as compared to vehicle treated controls. Interestingly, obese subjects showed a non-significant higher amount of multiple proliferating NK cells as compared to normal weight individuals (Figure 5A).

## Discussion

Excess bodyweight is associated with an increased risk of malignancy, e.g. esophageal adenocarcinoma and colon cancer [5,6]. However, the link between obesity and cancer susceptibility is still elusive. Leptin is a hormone secreted by adipocytes linking nutritional status with neuroendocrine and immune functions. The impact of leptin on human NK cells, part of the innate immune system, especially of a short- and long-term leptin exposure, needs to be further investigated.

In this study, we observed no difference of CD56^bright^ (expressed in high density on surface with activity in producing cytokines) and CD56^dim^ (expressed in low density with focus on cytotoxic features) NK cells between obese and normal weight individuals. NKT cells that have structural characteristics from NK as well as T cells were found to have a role in tumor immunity of mice without and following stimulation (such as αGalCer or IL-12 [36]). The reported anti-metastatic effect was dependent on IFN-γ production and NK cell activation through NKT cells [37,38]. Our data present significantly lower levels of NKT (CD3^+^CD56^+^) cells in obese subjects supporting the impaired protective activity due to obesity. Recent studies revealed type I and II NKT cells by different molecular markers [39]. Interestingly, patients with obesity (BMI > 40) and cancer had higher levels of type I NKT cells in the greater omentum compared to a lean control group [27]. Furthermore, clinical studies implied a correlation between type I NKT cell counts and prognosis for several human cancer types. By contrast, type II NKT cells were found to suppress anti-tumor immunity in several mouse models by producing IL-13 [39].

Like human T lymphocytes [18] and murine monocytes, NK cells express Ob-R. NK cells show increased levels of activated STAT3 as well as transcripted IL-2 and Perforin genes following leptin stimulation through Ob-Rb [20]. To evaluate the impact of endogenous high elevated leptin levels in obese subjects on the Ob-R expression we determined Ob-R on peripheral blood NK cells with flow cytometry. In obese subjects (with long-term endogenous leptin exposure) non-significant higher levels of Ob-R^+^ NK cells were found. However, we know from former studies in diet-induced obese rats [24] and human PBMCs [21] that Ob-R post receptor signaling is altered. Therefore we evaluated downstream signaling in response to an *in vitro* leptin challenge. Sanchez-Margalet et al. showed a transient activation of JAK2 in human PBMCs between 5 and 20 minutes after stimulation [38]. Post receptor signaling of Ob-R revealed lower levels in phosphorylation of JAK2 in PBMCs after leptin administration as compared to PBMCs of normal weight subjects confirming our data from previous studies.

Beside cytokine secretion, NK cells can induce apoptosis. TRAIL belongs to the TNF superfamily with its death receptor pathway for apoptosis. After stimulation with IFN-γ NK cells express TRAIL [30]. TRAIL is crucial for the IFN-γ dependent NK cell-derived protection from subcutaneous tumor growth as well as tumor metastasis in mice [29,30]. Moreover, mice infected with encephalomyocarditis virus showed earlier death after administration of anti-TRAIL mAb [40]. Interestingly, *in vitro* experiments showed that only inactivation of Fas ligand, Perforin and TRAIL decreases NK cell cytotoxicity against susceptible target cells [41]. With CD107a, there is an additional marker of NK cell activity. It has been demonstrated that CD107a expression correlates *in vitro* with lysis of target cells as well as cytokine production [33,42].

Here we present significantly higher levels of TRAIL^+^ NK cells in obese subjects. In contrast to expectations, the expression of CD178 and CD107a on NK cells revealed no difference between normal weight and obese individuals.

To further explain the discrepancy of normal OB-R^+^ NK cell counts and lower levels of p-JAK2 we investigated several NK cell activity parameters following leptin challenge. Wrann et al. showed diminished NK cell immune functions (such as IFN-γ secretion) after long-term incubation (72 h) with leptin. In contrast, short-term leptin stimulation revealed significantly higher functions of human NK cells [21] as well as in human NK cell lymphoma cell lines YT and NK-92 [20]. Takeda et al. demonstrated, that IFN-γ is essential for the suppression of subcutaneous tumor growth through TRAIL. Here we show comparable numbers of IFN-γ^+^ NK cells in normal weight and obese subjects. The amount of IFN-γ^+^ NK cells in normal weight subjects was significantly higher following short-term leptin challenge, whereas no effect was seen in the obese. This is in line with demonstrations of Wrann et al.: only short-term incubation (18 h) increased IFN-γ production of NK cells [21] and points towards a higher activity of innate immunity in normal weight individuals.

Beside cytotoxic activity, proliferation of NK cells is crucial for immune responses. In the present study, we observed no difference in the proliferation rate of human NK cells in both investigated groups. Interestingly, obese subjects had a non-significant higher amount of multiple proliferating NK cells as compared to normal weight subjects, suggesting a modified proliferation process in the obese.

Further studies with isolated NK cells and co-cultures with tumor cells are required to investigate the contribution of leptin and other adipocytokines on NK cell functions. Furthermore, other receptor pathways remain major goals to understand increased cancer susceptibility in obesity.

## Conclusions

The present study demonstrates significant lower TRAIL and p-JAK2 expression in NK cells from obese healthy humans. Chronically elevated endogenous leptin levels may be one mechanism for NK cell dysfunctions and consecutive increased cancer susceptibility in obese humans.

## Abbreviations

AU: Arbitrary units
BMI: Body mass index
FACS: Fluorescence-activated cell sorting
hrec: Human recombinant
JAK: Janus kinase
mAb: Monoclonal antibody
NK: Natural killer
Ob-R: Leptin receptor
PBMC: Peripheral blood mononuclear cells
PBS: Phosphate buffered saline
STAT: Signal transducer and activator of transcription
TBS: Tris-phosphatase-buffered saline
TRAIL: TNF-related apoptosis-inducing ligand
